# Consumers’ Perceptions of Coffee Health Benefits and Motives for Coffee Consumption and Purchasing

**DOI:** 10.3390/nu11030653

**Published:** 2019-03-18

**Authors:** Antonella Samoggia, Bettina Riedel

**Affiliations:** Department of Agro-Food Sciences and Technologies, Alma Mater Studiorum University of Bologna, Viale Fanin 50, 40127 Bologna, Italy; bettina.riedel@unibo.it

**Keywords:** consumer, behavior, perception, coffee, health, consumption motives

## Abstract

Coffee is popular worldwide and consumption is increasing, particularly in non-traditional markets. There is evidence that coffee consumption may have beneficial health effects. Consumers’ beliefs in the health benefits of coffee are unclear. The study aimed at analyzing consumers’ perceptions of coffee health benefits, consumption and purchasing motives of coffee consumers with positive perceptions of coffee health benefits, and willingness to pay for coffee with associated health claims. Data were collected through a face-to-face survey with consumers, resulting in a convenience sample of 250 questionnaires valid for data elaboration. Results were elaborated with factor analysis and logistic regression analysis. Findings revealed that a relevant minority of consumers believed that coffee could have positive health effects. The consumer with a positive perception of coffee health benefits is mostly male, young, works, is familiar with non-espresso-based coffee, consumes a limited amount of coffee (generally not for breakfast and often in social settings), and buys coffee at retail outlets. Consumers drink coffee for its energetic and therapeutic effects. Coffee consumption is still price-driven, but consumers are interested in purchasing coffee with associated health claims. There is the opportunity to improve the perception of coffee health benefits in consumers’ minds.

## 1. Introduction

Coffee is one of the most consumed beverages worldwide. Global coffee consumption is estimated to increase, particularly in non-traditional coffee drinking countries in Africa, Asia, and Oceania (+4.1%). Demand in traditional markets is estimated to grow by 1% in Europe and by 2.5% in North America [1]. Leading drivers for coffee market growth are innovations in out-of-home consumption, online commerce opportunities, and innovative brewed coffee beverage types [2]. Consumers are interested in coffee product quality and origin, as well as social, environmental, and economic sustainability [3].

Innovative coffee attributes related to the health properties of coffee could be a driver for coffee consumption [4]. Some researchers suggest that coffee might have the potential of a functional food thanks to its biochemical properties and the possible health benefits [5,6]. In particular, there is evidence that coffee consumption may have beneficial effects on non-communicable diseases (NCDs) [7]. This may contribute to the World Health Organization’s objective of reducing the relative risk of premature mortality from NCDs by 25% by 2025, by improving the modifiable risk factor of an unhealthy diet [8].

Consumers’ beliefs in the health benefits of coffee are unclear. Only 16% of U.S. consumers know about coffee’s health benefits, and 66% are prone to limiting their caffeine consumption [9]. Many European consumers are also confused about coffee’s impact on health, with 49% believing coffee has negative health effects [10]. On the other hand, consumption of green coffee-based beverages has become popular in recent years due to the belief in its beneficial antioxidant properties (e.g., chlorogenic acids, polyphenols) [5,11,12].

Coffee contributes to the daily intake of dietary antioxidants, more than tea, fruit, and vegetables [13]. A screening of the most consumed beverages for their bioactive non-nutrient contents identified instant coffee as the beverage with the highest total biophenol content [14]. Two other studies observed coffee to be the beverage with the highest total antioxidant capacity as compared to others like green and black tea and herbal infusions [15,16]. The biochemical composition of a cup of coffee depends on the degree of roasting, the type of bean (Arabica versus Robusta), and the coffee brewing method, including grind type [17,18,19]

There is little scientific knowledge on consumers’ attitude towards coffee health benefits. The perception of coffee’s health effects in consumers’ minds is unclear and has not been thoroughly researched. Past research studied consumer preferences and attitudes towards coffee attributes including sustainability, brands, coffee types, and motives for consumption like taste, energy, pleasure, socialization [20]. The paper aims to fill this gap in the literature and analyze the link between consumers’ coffee consumption behavior and their perception of coffee’s health benefits and risks. The research adds value to existing literature by analyzing what consumers perceive about coffee’s health effects. If coffee has positive effects on human health it would be important to educate consumers about the possible health benefits and the correct consumption of coffee. Therefore, it is important to first study the status of consumers’ perceptions about coffee’s health effects. Furthermore, this will allow for an exploration into whether there are marketing possibilities for coffee with health benefits considering the increasing consumption trend of healthy food.

In evaluating the healthiness of a cup of coffee it is important to consider that coffee drinking is a complex consumption behavior and that preferences and preparation methods are influenced by culture and tradition. To fully exploit coffee’s capability to impact on consumer food dietary lifestyle and health, there is need to better understand consumers’ coffee consumption habits, motives, and perception of coffee’s health benefits. Therefore, the objective of the research is to analyze consumers’ perception of coffee’s health benefits, consumption and purchasing motives of coffee consumers with positive perception of coffee health benefits, and willingness to pay for coffee with associated health claims.

Data was collected through a direct face-to-face survey with consumers using questionnaires with closed-ended questions. The structure of the paper is as follows. Section 2 provides a literature review of coffee consumption and purchasing motives and coffee and health, with a detailed review of the relevant literature on coffee’s effect on single health conditions. Section 3 describes data gathering and elaboration, and the data sample. Results are presented in Section 4. This section first discusses the results regarding consumers’ characteristics and perception of health effects of coffee, followed by insights on consumers’ perception of coffee health effects and motives for coffee consumption and purchasing, and concludes with analyzing consumers’ willingness to pay a price premium for coffee with associated health claims. Finally, the paper provides a discussion and conclusions on consumers’ perceptions of coffee’s health effects, profiling consumers according to their attitudes towards health coffee benefits. Section 6 puts the topic into the broader context of consumers’ increasing interest in healthy food and eating behavior, and reflects on marketing possibilities for coffee focusing on specific health benefits.

## 2. Literature Review

### 2.1. Coffee Consumption Motives

The scientific knowledge on motives and preferences of coffee consumption and purchasing behavior is fragmented. Past research focused strongly on a limited number of specific issues, particularly on aspects of sustainability and fair-trade labelling of coffee. Evidence from a recent systematic review of 54 papers on coffee consumer research [20] identified the leading motives for consumers’ coffee consumption and purchasing behaviors. Results suggest that there are several leading motives for coffee consumption: functional, taste and pleasure, habit, tradition and culture, and socialization. The main limiting factors for coffee consumption are a dislike of coffee’s taste and a belief in its possible negative health effects. The functional and the pleasure motives are the two leading drivers for coffee consumption and are of similar importance across cultures.

### 2.2. Coffee Purchasing Motives

Key coffee attributes that impact on consumers’ purchasing decisions are sustainability (including organic and fair trade), intrinsic quality attributes (e.g., roast degree, country of origin, variety), extrinsic attributes (packaging, brands), and coffee type (e.g., the espresso type includes black espresso and *macchiato*, that is, with a small amount of milk; other types include American long coffee (i.e., espresso topped with hot water), cappuccino, decaffeinated coffee, filter coffee, iced coffee, and coffee powder) [20]. A recent review on coffee purchasing motives did not identify studies that focused specifically on the relation between coffee price and consumer behavior [20]. There is limited research on consumer preferences for coffee’s intrinsic qualities. Preference for different intrinsic qualities depends on expertise and sensory skills of the consumer [21]. The untrained consumer has difficulties in distinguishing quality levels of coffee compared to an expert. The role of familiarity with the product is important in the assessment of its quality [22]. There is not much evidence on the role that extrinsic attributes and marketing play in buying decisions towards coffee; nonetheless, brands and labels are considered essential for the coffee industry. Research on brands, labels and packaging mainly concerns the willingness to pay for sustainability labels and the role of packaging and labels for the communication of sustainability information [23].

### 2.3. Coffee and Health

Consumers’ beliefs in health benefits or risks of coffee are inconclusive. For some the health benefit (e.g., anti-migraine effect) is a driver for consumption [24], others avoid coffee consumption for medical reasons like anxiety and insomnia [25], or because of the belief that coffee is generally bad for health [10]. Coffee drinking is not considered a health-oriented behavior, even if scientific evidence indicates that coffee can be part of a healthy diet [26,27]. The main health concerns arise with regard to the caffeine content of coffee [28]. Consumers see coffee mostly as a stimulant and are not informed about beneficial components and suggested health benefits [10].

Roasted coffee is a mixture of over 1000 bioactive compounds, with potentially therapeutic antioxidant, anti-inflammatory, antifibrotic, and anticancer effects [11,29]. Key active compounds are caffeine, chlorogenic acids, diterpenes, cafestol, and kahweol [7,30]. Coffee is rich in vitamin B3 and magnesium [6], and brewed coffee maintains the potassium concentration of the original seeds [31]. Caffeine is the most studied coffee component.

Scientific research has studied extensively the associations between coffee and all-cause mortality, cancer, cardiovascular diseases, neurological and gastrointestinal as well as liver systems, and all effects on pregnancy, with differing results over the years.

Current research concludes that coffee drinking is safe when consumed by healthy, non-pregnant women and adult persons in moderate quantity, equivalent to three to four cups per day, providing 300 to 400 mg/d of caffeine [7,26,28,32]. The largest reduction in relative risk of all-cause mortality was found with a consumption of three cups per day as compared with no consumption. Results suggest an inverse relationship between coffee drinking and all-cause mortality in men and women [7]. Daily coffee drinkers reduced their risk of dying prematurely compared with non-drinkers by 7–12% [33]. There were beneficial effects of coffee on cancer and cardiovascular diseases, as well as metabolic and neurological conditions [26]. Adverse effects of coffee drinking were mainly limited to pregnancy and to women at increased risk of bone fracture. Negative effects are mainly associated with caffeine rather than any other components in coffee [7,26]. Table 1 provides details on the studies focused on the effects of coffee on single health conditions.

The main limitation in drawing conclusions on coffee health associations is that existing evidence is observational and of lower quality. More research is needed with data from long-term randomized controlled trials [7,26,28].

## 3. Materials and Methods

### 3.1. Data Gathering

Data gathering was based on a direct face-to-face survey. Data was collected using questionnaires with closed-ended questions. The first question aimed at filtering interviewees so as to collect responses only from coffee consumers (i.e., those who generally drink coffee). The questionnaire includes five sections. Section 1 was on coffee consumption habits: types of coffee drunk (e.g., espresso, long coffee, cappuccino, decaffeinated, coffee powder, iced coffee, filter coffee); number of cups of coffee per day; occasions and places of consumption; companionship during consumption; consumption of other caffeinated drinks; type of coffee preparation; and outlets of coffee purchasing. Section 2 focused on motives of coffee consumption and purchasing (Table 2). Section 3 focused on the perception of health benefits of coffee. In particular, the first sub-section included questions aimed at eliciting the view of the consumers as to whether coffee consumption can bring health benefits, can reduce diseases, can be a functional beverage for human wellness, and has nutritional properties that can improve human health. These items are based on coffee health impact literature review, past research studies exploring consumers’ perception of food healthiness [4,9,66,67,68,69,70,71], and the European Food Safety Agency food health and nutrition claims [72]. The second sub-section asked consumers’ opinions on the effects of moderate coffee consumption on diminishing the risk of diseases and on influencing a number of physical effects based on scientific-tested studies (Table 1). Then, the third sub-section asked if consumers thought that there was a gender difference in terms of coffee consumption with respect to health, and whether decaffeinated coffee had different health impact compared to caffeinated coffee. These items are based on a coffee health impact literature review. Section 2 and Section 3 asked the respondents to rate each question using a 5-point Likert scale of agreement/disagreement (1: “totally disagree” to 5: “totally agree”, with scale end values anchored to interpretations), or with other responses options (e.g., “yes”/”no”) as reported in the Table notes.

In the fourth section respondents were asked to state their willingness to pay (WTP) for the most common type of coffee product, the coffee brick pack. Only participants that more frequently bought this type of coffee were considered in the analysis. Participants’ WTP was assessed by applying the multi price list (MPL) in a hypothetical setting method, widely adopted in experimental economics [73,74,75]. This mechanism has the great advantage of being transparent and very simple to understand for participants. The minor disadvantage is the interval response with a psychological bias toward the middle of the list [76]. Before eliciting their WTP, participants were provided with a reference price for the product type that was identified based on current retailer prices. The price premiums went from €0.10/brick to €1.50/brick, with 15 price premium options with a €0.10 difference. Section 6 gathered information on the socio-demographic profiles of the respondents.

The questionnaire was tested in trial face-to-face interviews and the items identified as unclear or not important were revised. Interviewers carried out 272 interviews. Data cleaning led to the definition of a convenience sample of 250 questionnaires for data elaboration. The places of interviews were retail outlets, coffee shops, bars, and malls. Interviews were carried out from April to July 2018. At the beginning the interviewer declared the interview was part of a university study, wore a badge with name and university affiliation, and proceeded with the interview if the respondent agreed to participate in the research. The time necessary to carry out each interview was around seven minutes. No reward or token was awarded. Data were collected with the support of the Qualtrics survey program by uploading the answers gathered during the face-to-face interviews.

### 3.2. Data Elaboration

Data elaboration followed different phases. First, data elaboration calculated the consumers’ level of perception of coffee health benefits. The level of perception was calculated as mean value of the first sub-section items belonging to Section 3, that is, whether consumers agreed that coffee consumption could bring health benefits, reduce diseases, be a functional beverage for human wellness, and have nutritional properties that can improve human health. The mean values of positively versus negatively inclined consumers were cross-checked with the analysis of variance (ANOVA). The levels of perception of positively versus negatively inclined consumers were cross-analyzed with consumers’ socio-economic characteristics and coffee consumption habits, and tested using the chi-squared test.

Second, the research identified the existing latent factors in consumers’ coffee consumption and purchasing motives, with the support of two factor analyses. Two separate factor analyses were run, one for coffee consumption motives, and one for the coffee purchasing motives in order to highlight possible different habits in the consumers’ approaches to coffee. The principal components method (PCA) and Varimax rotation (Eigenvalue criterion being higher than 1) were applied.

Third, the factors were used in the logistic regression (enter method), carried out to explore the relationship between consumers’ perceptions of health benefits of coffee and their consumption and purchasing motives. The factor variables were also checked for the multicollinearity analysis, to verify the possibility that one variable is a linear function of the other. Multicollinearity has been tested through tolerance and variable inflation factors (VIFs) [92]. Omnibus tests of model coefficient were analyzed to test the level-of-fit of the model. Model variance with Nagelkerke was considered. Finally, the research calculated the WTP and cross-analyzed values with socio-economic characteristics of the consumers. Data elaboration was carried out with the support of SPSS (version 21).

### 3.3. Sample

Out of the 250 respondents, the majority were women, and about half had an academic degree (Table 3). There was a majority of people working, and a generally low or medium family income. The age was well distributed, as 55.2% of the respondents are aged younger than or equal to the average age, that is, 40.97 years (maximum age is 85 and minimum age 18).

## 4. Results

### 4.1. Consumers Characteristics and Perception of Health Effects of Coffee

A relevant minority of consumers (25%) thought that drinking coffee could have positive effects on health (Table 4). The average value of the perception on coffee health benefits of the positively inclined consumers was fairly high (3.7). The analysis of consumers’ socio-economic characteristics, coffee consumption, and purchasing habits of the positively versus the negatively inclined consumers showed interesting elements (Table 4). A higher percentage of men (31%), of younger (30.4%), and of working (27.2%) consumers had a positive perception of the health effects of coffee consumption compared to female, older, and not working consumers. The level of education was not an explanatory characteristic for the perception of health effect of coffee consumption. There were more consumers that tended to drink non-espresso based coffee (36.2%), that consumed from one to two cups of coffee per day (32.5%), that never or rarely drank coffee for breakfast (34.3%), and that bought coffee in big retailer chains (27.9%) that had a positive perception of coffee health benefits. A chi-squared *p*-value confirmed the results. Other data support that positively inclined consumers tended to drink coffee with other people (28.5%), and that they did not to have coffee as a break (29.4%) or after lunch (28.1%).

These results suggest that consumers positively inclined towards coffee health benefits are more likely to be male, young, and working, tending to appreciate non espresso-based coffee, consume in limited amounts and in social settings, and not usually consuming in the morning. They are more likely to purchase it in common outlets, probably with other food items.

**Figure 1:**
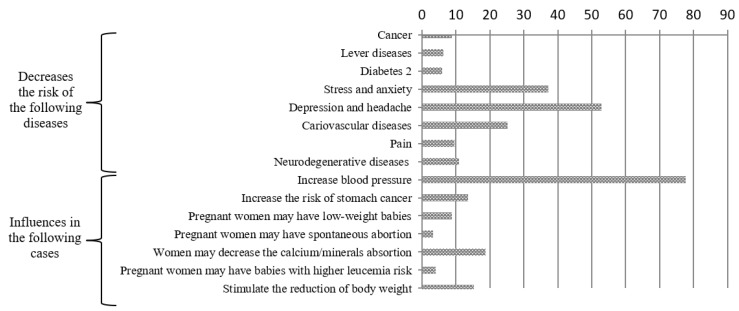
Consumers’ perception of health effect of coffee consumption (%). Note: Consumers’ response options were “yes”/”no” for each item. Therefore, the figure shows that around 80% of respondents thought that drinking coffee increased blood pressure.

Consumers are better inclined towards a limited number of benefits of coffee consumption (Figure 1). In particular, almost 80% of consumers believe that drinking coffee increases blood pressure, more than half think that it decreases depression and headache, one-third that it decreases the risk of stress and anxiety, one-fourth that it decreases the risk of cardiovascular diseases, and one-fifth that it impacts on women’s capability to absorb calcium and minerals and stimulates the reduction of body weight. Consumers do not acknowledge other medically tested effects on pregnant women, diabetes, liver, cancer, neurodegenerative diseases, and pain.

Moreover, 61% of consumers believe that the correct number of cups of coffee per day is between three and four. According to scientific studies, this is the recommended quantity (equivalent to 300–400 milligrams of caffeine per day) [7,26,32]. Therefore, the vast majority has an adequate knowledge of the daily quantity of coffee to be consumed. Around 35% of consumers think that between one and two cups is adequate, values lower than the threshold set by scientists, thereby showing some skepticism towards coffee impact on health. Moreover, 84% of consumers think that the effect is similar in men and women, and 80% that decaffeinated coffee has a similar impact to caffeinated coffee on human health. These results support that consumers have adequate knowledge on the quantity to be consumed, the effects on gender, and the types of coffee, fairly in line with scientific evidence [7,26,32]. There is no evident misconception of the effects of coffee on health.

### 4.2. Consumers’ Perception of Coffee Health Effect and Motives for Coffee Consumption and Purchasing

The two factor analyses on consumers’ coffee consumption and purchasing motives identified seven main components (Table 5 and Table 6). Four components derive from the factor analysis on the initial 12 items on coffee consumption motives, and three components derive from the factor analysis on the initial 13 items on purchasing motives. The second factor analysis was tested until all identified components had satisfactory internal consistency values. This lead to delete three items. In both factor analyses items were loaded into single factors, with factor loadings above 0.585. The Kaiser–Meyer–Olkin measure of sampling adequacy and Bartlett’s test of sphericity were calculated to assess the appropriateness of the data for factor analysis. The Kaiser–Meyer–Olkin index was 0.649 in the coffee consumption motives PCA and 0.660 in the coffee purchasing motives PCA. Bartlett’s tests of sphericity were highly significant (0.000). The cumulated variance values explained by the factors were respectively 66.2 and 66.3. Elaboration results confirmed the data appropriateness. The values of the factors were calculated based on the mean of the items loading into the single factors.

The internal consistency and convergent and discriminant validity of each component was verified (Table 5 and Table 6). The internal consistency of each set of items was measured using Cronbach’s alpha and composite reliability (CR). Alpha component values were from 0.633 to 0.771, and CR values were from 0.77 to 0.88 in the first factor analysis. In the second factor analysis, alpha component values were from 0.675 to 0.836 and CR values were from 0.81 to 0.94. Values were satisfactory and acceptable [93,94]. The average variance extracted (AVE) provides a measure of convergent validity, and ranged from 0.504 to 0.696 in the first factor analysis and from 0.510 and 0.776 in the second factor analysis. These were satisfactory as above the 0.50 threshold [95]. To confirm discriminant validity, the square root of each construct’s AVE was calculated to ensure it was greater than its bivariate correlation with other constructs in the model. This led to adequate outcomes. The results confirm the reliability and validity of the research components.

The factors were labeled according to coffee consumption and purchasing motives associated with the statements. Coffee consumption is driven by four main factors. The most important factor is the habit and pleasure of drinking it (3.1). This connects to the organoleptic characteristics that are coffee smell and taste, family traditions and habits, and the emotions and moods created by coffee. The energetic physical and mental awakening power of coffee is as important as its role in having a break during the day and socializing at work (2.7). The fourth motive for drinking coffee is its therapeutic impact, that is, the capability of coffee to help digestion, increase blood pressure, and alleviate headaches (1.7). Coffee purchasing is driven by three main motives. The main driving element is the price, that is promotion and value for money (3.3). Another key aspect is the declared aroma, recipe, level of roasting, and intensity (3.2). The coffee sustainability (1.8) does not strongly influence consumers’ coffee purchasing. In synthesis, consumers have a hedonistic approach towards coffee, focused on its taste, smell, and family habits and culture. Their consumer behavior is also driven by utilitarian reasoning, focused on price. In addition, coffee is drunk for its relevant socializing and energetic power.

There is a statistically significant relationship between consumers’ perception of coffee health benefits and motives for coffee consumption and purchasing (Table 7 and Table 8). The VIF values were between 1.020 and 1.401, and the lowest tolerance value was 0.714. Therefore, there was no multicollinearity between variables. The significant relation is between the perception that coffee can have health benefits, and the following motives of coffee experience: habit and pleasure (0.017), aroma (0.048), and price (0.058). The significant relation is in some cases an unpredicted direction. If the consumers believe in the coffee health benefits, they tend not to drink it as a habit or for pleasure or consume coffee for its aroma. Moreover, the positively inclined consumers believe price is a motive of coffee purchasing. Results are confirmed by *p*-values.

These results suggest that if consumers drink coffee for the pleasure of it, out of family and traditional habits, and because of the taste and coffee roasting/recipes, then they are distant from the idea that coffee may have a positive health impact. If their coffee purchasing experience is influenced by the product price, then they are sensitive to coffee’s health impact. If coffee purchasing and consumption are not driven by hedonism and traditional routine and are not emotional, then their perception is better inclined towards new features of coffee.

### 4.3. Consumers’ Willingness to Pay a Price Premium for Coffee Health Benefits

The vast majority of consumers (74%) is willing to pay a price premium for coffee with health benefits (Table 9). Given that the average price is around €2.75/brick pack, a €1.03 average price premium is equivalent to +37% (average price is €2.78/250 g brick pack, equivalent to €11/kg) [96]. The price premium is significant. There are variations among the different socio-economic groups of consumers. The highest price premium (between €1.00 and €1.50) would be paid mostly by older (62.9%) and higher income consumers (17.5%). A higher percentage of women (70.4%) are favorable towards fairly high coffee price premiums (between €0.51 and €1.00).

## 5. Discussion

The debate over coffee’s effects on the human body has gone through various stages, with recommendations aimed at promoting or avoiding coffee consumption. The history of coffee started in the 15th century [97]. Its consumption first grew in Arabic countries and then expanded to Persia, Egypt, Syria, and Turkey. It was known as “wine of Araby”, and drunk as a substitute for alcohol, which was prohibited according to the Islamic religion. In the 17th century coffee arrived in Europe (e.g., Italy, England, France, Austria). Consumers increasingly drank it in coffee houses that become competitors for pubs, with coffee becoming a substitute for beer and wine. During the 18th century it became common in North America, and then, thanks to the optimal weather, it was cultivated in South America. Brazil is currently the most significant coffee-exporting country. During its long history, coffee has been criticized for various reasons: because it was considered to stimulate critical thinking (Mecca), because it was considered Satanic (Italy), because it was considered as a toxic substance used to bring about death (unsuccessfully) (Sweden), and because it threatened beer consumption and therefore local agricultural production (Prussia) [97,98]. As history shows, coffee consumption and the beliefs in its nutritional properties have always been intertwined. Coffee properties perceptions have often shaped coffee consumption and purchasing habits, including preparation methods, favorite types of coffee, and places of consumption and purchasing.

The present research paper provides valuable insights on consumers’ perception over coffee health effects, and profiles coffee consumers’ characteristics based on their positive or negative attitudes towards coffee health effects. There are a number of results that highlight consumers’ socio-economic characteristics and coffee consumption habits, consumers’ motives for coffee consumption and purchasing, and consumers’ interest in coffee with associated health claims.

The present research shows that men are more positively inclined towards coffee health benefits as compared to women. Women appear more skeptical, whereas a higher percentage of men already believe that drinking coffee benefits their health. Considering women’s general strong propensity towards healthy food [99], coffee with certified health claims may lead women to have a more positive inclination towards it. Moreover, the consumer with a positive attitude towards coffee health benefits is fairly young, works, and has a habit of drinking coffee in social occasions, in limited quantity, and in various preparations, not necessarily espresso. This approach to coffee drinking is in line with the most recent coffee consumption trends. Recent studies support that there is an increasing number of people drinking coffee, with interest in gourmet coffee, new types of coffee (e.g., frozen blended coffee drinks, nitro coffee, and cold brew), out-of-home consumption, and lower appreciation for cafe moka [9]. Moreover consumers believe coffee has some effects on the human body (e.g., blood pressure, depression, headache, stress and anxiety, body weight). This suggests that there are no specific misconceptions over coffee, but consumers are still not fully aware of coffee’s nutritional potential and health impacts.

Results on the motives for coffee consumption support that the energy coffee provides is the key health effect consumers aim for. Coffee drinkers expect improved alertness and higher physical and mental performance [24,25,77,78]. There are motives for coffee consumption that differ among the positively and negatively inclined consumers with respect to coffee’s health benefits. The positively inclined consumer to a certain extent values coffee for its aroma, pleasure, habits, and socialization. This is a relevant difference compared to past studies that supported taste as the main motive for coffee drinking [25,77,78,79]. In consumers, coffee evokes feelings of pleasure and comfort during the drinking experience [77,78,79]. The wide audience of coffee consumers gives particular importance to coffee habit and family traditions that influence preferred occasions, locations, and types of coffee consumption [24,25,82]

Despite the fact that positively inclined consumers drink coffee with others to have a break, socialization is not a key motive. This approach brings a distinguishing interpretation with respect to past studies. These studies suggest that drinking coffee is a way to socialize and be part of a group [25,77,79,82]. In synthesis, the energizing effect is what the consumer aims for. The consumer aims for a functional drink with a clear mental- and body-stimulating function. This is the same consumer objective for soft drinks and energy drinks.

Results on the motives of coffee purchasing support that for the positively inclined consumer, price is a significant attribute. The consumer is influenced by extrinsic coffee attributes. Coffee purchasing is to a certain degree driven by aroma, coffee recipe, brand, information, and emotions, but rather by rational and economic elements. Therefore, for these consumers messages focused on health claims that give value to the money spent may be important for coffee consumption and purchasing. Past studies found that the use of texts, brands, and metaphorical images on coffee packaging moderately influenced product expectations, intrinsic quality perception, and purchase intention [89]. Brand identification is especially important in the coffeehouse market [87,88,89,90]. Drinking a specific coffee brand (e.g., Starbucks) represents a status symbol and way of life for consumers [87,88].

Sustainability is one of the most studied subjects in consumer purchasing research on coffee [20]. Present and past research results suggest that aroma, price, and promotions are more important factors as compared to sustainability [85]. Only consumers with a strong attitude towards sustainability gave more importance to the sustainability claims over hedonic attributes and were willing to pay more for sustainably produced coffee [84,86,100].

The present research on consumers’ interest in the economic investment over coffee products with health claims further highlights the importance of price in coffee purchasing. Results show that price is an important element for all consumers and that coffee is mostly purchased from large retailers. The importance of price in coffee purchasing shows that coffee is still a rather undifferentiated commodity. Consumers with positive attitudes towards coffee’s health benefits give particular importance to price. Moreover, consumers are generally willing to pay higher prices for coffee with health claims. This is suggested for both positively and negatively coffee health-oriented consumers. In particular, women and consumers with higher monetary resources are more favorable towards healthy food. This is consistent with past research results [101,102,103].

The willingness to pay for coffee with innovative attributes is confirmed by the market expansion of coffee capsules. Capsules have been successful thanks to the low cost of machines, the ease of use, the practicality of packaging, and effective marketing communication campaigns [96,104]. This success was achieved despite the high price, with consumers willing to pay up to five times more than coffee powder brick (around €55/kg for coffee capsules). This market phenomenon has been disruptive for the coffee market. It contributed to stopping the price competition that excessively lowered the price of the powder coffee brick, coffee quality, and the capability for investing in coffee research and development as well as innovations.

## 6. Conclusions

Consumer attitudes toward food products determine consumption behavior more than knowledge. Attitudes and perceptions influence dietary behavior intentions [105]. Results from the current study on coffee consumers’ consumption and purchasing habits can contribute to a better understanding of food lifestyle decisions. The integration of knowledge of nutritional qualities with knowledge of consumers’ expectations and perceived food qualities allows for addressing possible misconceptions and more effectively defining food consumption and purchasing behavior recommendations.

There is an expanding consumers’ interest for healthy food. Consumers are increasingly aware of the impact food has on body functions [69,71,106]. Coffee consumption has often been negatively criticized for its health effect. Recent studies show that coffee can have positive health effects, but consumers are still cautious on drinking coffee. The coffee image is of a drink with a health impact, but not necessarily positive, and not based on the latest science-based outcomes. Coffee is used for its energetic and therapeutic effects. Together with other energy drinks, it is increasingly used as a substitute for soft drinks. Coffee is a drink with some advantages. It is naturally low in calories if drunk “black”, and it is a drink good for socializing. Coffee chains are expanding. Soft drinks companies are increasingly interested in developing their business to include coffee shop chains [107].

The coffee market is very dynamic, and consumers are increasingly interested in artisanal coffee and small coffee breweries. Drinking coffee is already acknowledged as a pleasure. The aspects of aroma, taste, smell, and occasions of consumption are still crucial. However, there is space to improve perceptions of scientifically-based health benefits. To increase awareness and improve knowledge among consumers, coffee marketing strategies could focus more on health benefits and nutritional values of coffee [4,66,108] in addition to the other positive characteristics consumers already associate with coffee. As a result, coffee consumption could be marketed as being pleasant and healthy at the same time.

There are already examples for market trends and innovations focusing on the functional and health aspects of coffee. Ready-to-drink (RTD) coffee (packaged liquid coffee designed to be consumed when opened without any additional steps) is interpreted as a clean functional beverage category and a healthier alternative to soft drinks. The RTD coffee segment is expected to grow due to global trends in the coffee sector: worldwide coffee culture growth, active on-the-go-lifestyle, and investments by major players [109]. Some coffee brands already use health focused strategies for coffee marketing (RTD and ground coffee). RTD cold brew coffee is marketed as a sugar and fat-free alternative to traditional energy drinks [110] or as a probiotic cold brewed coffee supporting digestive and immune health [111]. There are examples for a prebiotic fiber-enriched ground coffees with digestive health benefits [112] and for antioxidant-enriched ground coffees [113].

The discussion whether coffee can be claimed as an actual functional food is ongoing and there is not enough long-term evidence that coffee can prevent disease. Therefore coffee consumption for health reasons requires further scientific evidence before being recommended and promoted [7,28,114].

### Limitations and Future Research

There are some study limitations. Results come from a convenience sample, focused on Italian consumers. Future studies may aim for samples with statistical representativeness and compare perceptions of consumers living in different countries. Coffee consumption behavior is related to various countries’ consumption traditions and habits, and cross-country analysis may bring a more comprehensive perspective. Furthermore, considering the fast development in coffee consumption habits, future studies may focus the analysis on consumers that specifically favor coffee consumption out-of-home or specific coffee types preparations, such as filter, capsules, and powder. Future studies may also test consumers’ WTP for different combinations of coffees with associated health claims such as disease reduction and health-promoting effects. Finally, future studies may explore coffee consumption motives within the dietary lifestyle, so as to provide sound information on the food behavior of coffee consumers for nutritionists and doctors.

## Figures and Tables

**Table 1:** Effects of coffee on single health conditions.

Cardiovascular disease	Habitual coffee consumption was consistently associated with a lower risk of cardiovascular diseases mortality [7,31]. Compared to non-coffee drinkers, risk was reduced by 19% and the largest reduction in relative risk was found at three cups per day [7,34,35]. Coffee consumption may have a protective effect on the risk of stroke [36,37], especially in women [38]. Research found a 30% lower risk of mortality from stroke of coffee consumers compared to non-drinkers [7]. The reduced risk for cardiovascular conditions is related to the antioxidant effects of coffee [26,39].
Type-2 Diabetes	Polyphenolic coffee compounds have beneficial effects on insulin and glucose metabolism [26,31]. Coffee consumption was associated with a lower risk of developing type 2 diabetes [7], with a stronger effect for women [40]. An intake of three to four cups of coffee/day seems to lower the risk by 25% compared to no coffee or less than two cups a day [34,41,42]. A meta-analysis concluded that the risk to develop type 2 diabetes decreased by 6% for each cup-per-day increase in consumed coffee [43].
Liver Conditions	Coffee consumption is related to a lower risk of developing several liver conditions [44,45]. There is an inverse association between coffee consumption and liver cancer [46,47]. Phenolic compounds, melanoidins, and caffeine are responsible for antioxidant effects in the liver [26].
Neuro-degenerative disorders	Lifelong, regular and moderate coffee consumption might have a beneficial effect on physiological, age-related cognitive decline/dementia [48,49], Parkinson’s disease [50,51], and Alzheimer’s disease [52,53]. The potential beneficial effects of coffee on mental health seem to be related to the neuroprotective effect of caffeine [26,50].
Depression and anxiety	Caffeine and other polyphenolic compounds of coffee have been associated with positive effects on mental health, for example behavior, mood, depression, and cognition [7,54]. On the other hand, high caffeine consumption is associated with anxiety and nervousness. Positive effect on mood is influenced by time of consumption, being highest in the late morning [55]. Caffeine seems to be more beneficial for habitual consumers [56]. Coffee consumption had a consistent association with lower risk of depression [26,57] and to relieve depressive symptoms [58].
Cancer	The International Agency for Research on Cancer (IARC) evaluated in 2016 a database of 1000 observational and experimental studies on coffee and cancer and concluded that there are no clear associations between coffee drinking and cancer at any body site. Coffee was classified as an agent “not classifiable as to carcinogenicity to humans”. There is evidence for a lower risk of cancer in high versus low coffee consumption [7]. Phytochemical compounds in coffee (diterpenes, melanoidins, polyphenols) may have beneficial effects at the cellular level, for example inhibiting oxidative stress and damage [26]. There is evidence that coffee intake is associated with a reduced risk of certain cancers [30,59].
Lung and gastric cancers	An adverse effect of coffee consumption has been seen in an increased risk of lung and gastric cancers. In this case, it is important to consider the potentially modifying effect of associated smoking habits. A subgroup analysis showed that the association was significant only in studies that did not adjust for smoking behavior [7,26].
Blood pressure	Coffee consumption has been associated with a rise in blood pressure [26]. Coffee intake raises blood pressure in non-coffee-drinkers, but not in habitual coffee drinkers. On the other hand it was observed that the antioxidant compounds of coffee might counteract the effects of caffeine in raising blood pressure [26]. Research results are conflicting and the association between coffee consumption and blood pressure remains unclear [60].
Pregnancy	Negative associations of coffee and caffeine intake were mostly pregnancy-related (low birth weight, pregnancy loss, preterm birth, childhood leukemia) [7,26,61,62]. The European Food Safety Authority (EFSA) [32] recommends that a moderate caffeine intake of 200 mg/day does not increase the risk of any pregnancy-related complication. Still, the association between coffee/caffeine and reproductive health outcomes needs further investigation as available data are insufficient and the role of confounding (e.g., diet, smoking etc.) factors is unclear [61].
Bone fracture	A negative association between coffee consumption and bone fracture was seen in women [7]. A 14% higher risk was found in high versus low coffee consumption [63]. The increased risk in women seems related to caffeine and its potential influence on calcium absorption [64] and bone mineral density [65]. The systematic review by Wikoff et al. [28] concludes that a caffeine intake of 400 mg/day was not associated with negative effects on fracture, bone mineral density, and calcium metabolism.

**Table 2:** Literature references for studied items in the questionnaire.

Item	Literature References
Functional (awakening and attention, physical energy)	[24,77,78,79]
Sensory (taste, smell)	[25,77,78,79,80]
Pleasure (mood and emotion, comfort, relaxing)	[77,78,79]
To socialize (with family, friends, coworkers)	[25,79,80,81,82]
To have a break	[10,25,77]
Health (digestion, against headache, increase blood pressure)	[24,25,77,81]
Family tradition and culture	[24,25,82]
Habit	[24,81,82]
Price, promotion, value for money	[23,83,84]
Coffee roast, coffee recipe, intensity and taste information	[2,22,80]
Country of origin	[20,80,85,86]
Sustainability (fair-trade, organic)	[23,84,85,86]
Brand knowledge, packaging, advertising	[83,87,88,89,90]
Expert recommendations	[21,91]

**Table 3:** Sample characteristics.

Gender	%
Women	66.4
Men	33.6
Total	100.0
LEVEL OF EDUCATION	
No academic degree	51.0
With academic degree	49.0
Total	100.0
AGE	
Below or equal to average age	55.2
Above average age	44.8
Total	100.0
EMPLOYMENT STATUS	
Working	80.8
Not Working	19.2
Total	100.0
LEVEL OF FAMILY INCOME	
Low and medium income (up to €55,000/year)	87.3
High income (above €55,000/year)	12.7
Total *	100.0

* 39.1% did not respond to this question (“I do not know” or “I do not want to respond”).

**Table 4:** Consumers’ perceptions of health effect of coffee consumption and consumers’ characteristics.

	**Negative Perception %**	**Positive Perception %**	**Total**	**ANOVA**	***p*** **-Value**
Total ^a^	75.2	24.8	100		
Perception of health effect of coffee (average) ^a^	2.29	3.70	2.91	0.000	***
Standard deviations	0.500	0.484	0.762		
Socio-economic characteristics					
	**Negative Perception %**	**Positive Perception %**	**Total**	**Pearson’s chi-squared**	***p*** **-Value**
Gender					
Men	69.0	31.0	100	0.075	*
Women	78.3	21.7	100		
Age					
Below equal to average age	69.6	30.4	100	0.015	**
Above average age	82.1	17.9	100		
Level of education					
No academic degree	72.0	28.0	100	0.153	
Academic degree	78.4	21.6	100		
Working condition					
Working	72.8	27.2	100	0.047	**
Not working	85.4	14.6	100		
Consumption and purchasing habits					
Type of coffee most frequently drunk ^b^					
Espresso	77.8	22.2	100	0.038	**
Non espresso-based coffee	63.8	36.2	100		
Frequency of consumption					
One to two cups of coffee/day	67.5	32.5	100	0.038	**
Three or more cups of coffee/day	78.8	21.3	100		
Companionship in consumption					
On my own	78.7	21.3	100	0.121	
With others	71.5	28.5	100		
Place of consumption					
At home	75.5	24.5	100	0.527	
Out of home	75.0	25.0	100		
Method of preparation most frequently adopted ^c^					
Moka pot	76.6	23.4	100	0.409	
Capsules	74.4	25.6	100		
Consumption of caffeine ^d^					
Low/medium caffeine consumption	75.7	24.3	100	0.497	
High caffeine consumption	74.8	25.2	100		
Coffee Consumption for breakfast					
Never/rarely	65.7	34.3	100	0.098	*
Often/always	77.1	22.9	100		
Coffee Consumption as a break					
Never/rarely	70.6	29.4	100	0.106	
Often/always	78.4	21.6	100		
Coffee Consumption after lunch					
Never/rarely	71.9	28.1	100	0.228	
Often/always	77.0	23.0	100		
Coffee Consumption after dinner					
Never/rarely	76.0	24.0	100	0.382	
Often/always	73.2	26.8	100		
Place of purchasing					
Big retailer	72.1	27.9	100	0.096	*
Small retailer	82.5	17.5	100		

Note: *, **, *** Significant at *p* <0.10; *p* <0.05; *p* <0.01; ^a^ Based on the average value of coffee health impact perception. Negative and neutral coffee health impact (below or equal to 3); Positive coffee health impact (above 3). ^b^ “Espresso” type includes black espresso and *macchiato*, that is, with a small amount of milk; “Other types” include American long coffee (espresso topped with hot water), cappuccinos, decaffeinated coffee, filter coffee, iced coffee, and coffee powder. ^c^ The moka coffee pot is the most common coffee brewing technique in Italy. This results includes only the moka coffee pot and capsules as they were the most frequently ticked answers (94%). ^d^ Other sources of caffeine consumption, in addition to coffee, are: tea, energy drinks, coke, other caffeine drinks. Low/medium caffeine consumption has values of 1, 2, 3. High caffeine consumption has values of 4 and 5 in a 5-point Likert scale where 1 is “never” and 5 is “always”.

**Table 5:** Factor analysis on motives for coffee consumption and convergent validity and discriminant validity for each construct.

	**Habit and Pleasure**	**Social**	**Therapeutic**	**Energy**
Awakening and attention				0.880
Physical energy				0.882
Cronbach’s alpha 0.742				
Habit	0.669			
Mood and emotion	0.585			
Family tradition and culture	0.693			
Smell	0.814			
Taste	0.786			
Cronbach’s alpha 0.771				
To have a break		0.841		
To socialize		0.798		
Cronbach’s alpha 0.665				
Digestion			0.651	
Against headache			0.798	
Increase blood pressure			0.717	
Cronbach’s alpha 0.633				
Variance explained (%)	21.97	14.12	13.91	13.90
Mean value of factors	3.1	2.7	1.7	2.7
Convergent validity and discriminant validity				
	**Habit and Pleasure**	**Social**	**Therapeutic**	**Energy**
Habit and pleasure	*0* *.510*			
Social	0.324	*0.672*		
Therapeutic	0.092	0.187	*0.525*	
Energy	0.273	0.194	0.173	*0.776*
Composite reliability	0.84	0.81	0.77	0.88

Note: Diagonal data (in italics) represent Fornell and Larcker’s average variance extracted (AVE). Subdiagonal represent the inter-construct correlations.

**Table 6:** Factor analysis on motives for coffee purchasing and convergent validity and discriminant validity for each construct.

	**Price**	**Sustainability**	**Aroma**
Price	0.902		
Value for money	0.859		
Promotion	0.842		
Cronbach’s alpha 0.836			
Coffee recipe			0.663
Coffee roast			0.775
Brand knowledge			0.641
Intensity and taste information			0.752
Cronbach’s alpha 0.675			
Country of origin		0.735	
Fair-trade		0.910	
Organic		0.848	
Cronbach’s alpha 0.790			
Variance explained (%)	24.21	22.02	20.11
Mean value of factors	3.3	1.8	3.2
Convergent validity and discriminant validity			
	**Price**	**Sustainability**	**Aroma**
Price	*0.517*		
Sustainability	0.069	*0.696*	
Aroma	0.017	0.101	*0.504*
Composite Reliability	0.94	0.88	0.81

Note: Diagonal data (in italics) represent Fornell and Larcker’s average variance extracted (AVE). Subdiagonal represent the inter-construct correlations.

**Table 7:** Logistic regression on the relationship between consumers’ perception of coffee health benefits and motives for coffee consumption and purchasing.

	B	S.E.	Wald	Sig.		Exp(B)	Tolerance	VIF
Habit/pleasure	−1.037	0.433	5.744	0.017	**	0.355	0.980	1.020
Social	−0.359	0.440	0.664	0.415		0.699	0.912	1.097
Energy	−0.510	0.838	0.370	0.543		0.601	0.714	1.401
Price	0.706	0.373	3.585	0.058	*	2.027	0.961	1.041
Sustainability	−0.627	0.631	0.987	0.320		0.534	0.755	1.325
Aroma	−0.816	0.412	3.925	0.048	**	0.442	0.972	1.028
Constant	2.099	1.403	2.236	0.135		8.155		

Dependent variable: level of coffee health benefit perception—(0) negative and neutral (average value below or equal to 3) vs. (1) positive (average value above 3). Note: *, ** significant at *p* < 0.10; *p* < 0.05. Omnibus tests: 0; VIF: between 1.020 and 1.041; Nagelkerke R-square: 0.313. The limited number of consumers with positive perceptions of coffee’s health benefits and with consumption behavior driven by therapeutic motives (one consumer) suggests not including the therapeutic component in the regression exercise. VIF: variable inflation factor.

**Table 8:** Relationship between consumers’ perception of coffee health benefits and motives for coffee consumption and purchasing, with chi-squared results

		Consumers Perception of Coffee’s Health Benefits (%)		Total	Chi-Squared	
		Negative	Positive			
Habit/pleasure	Negative	63.7	85.4	75.9	0.000	***
	Positive	36.3	14.6	24.1		
Social	Negative	72.2	85.7	76.8	0.022	**
	Positive	27.3	14.3	23.2		
Therapeutic	Negative	76.1	91.7	77.0	0.192	
	Positive	23.9	8.3a	23.0		
Energy	Negative	76.3	72.2	76.0	0.442	
	Positive	23.7	27.8	24.0		
Price	Negative	82.2	71.0	76.2	0.031	**
	Positive	17.8	29.0	23.8		
Sustainability	Negative	76.5	82.6	77.2	0.361	
	Positive	23.5	17.4	22.8		
Aroma	Negative	65.6	87.9	78.5	0.000	***
	Positive	34.4	12.1	21.5		

Note: **, *** significant at *p* < 0.05; *p* < 0.01.

**Table 9:** Willingness to pay a price premium for coffee with associated health claims (%).

	Yes, I Am Willing to Pay a Price Premium 73.6%		
	From €0.10 to €0.50	From €0.51 to €1.00	From €1.01 to €1.50
All consumers (average €1.03)	17.2	28.4	28.0
Men	33.9	29.6	37.1
Women	66.1	70.4	62.9
Total	100.0	100	100
Below equal to average age	62.4	62.0	37.1
Above average age	37.6	38.0	62.9
Total	100	100	100
Low and medium income	91.7	92.1	82.5
High income	8.3	7.9	17.5
Total	100	100	100
